# Targeted Next Generation Sequencing in Patients with Inborn Errors of Metabolism

**DOI:** 10.1371/journal.pone.0156359

**Published:** 2016-05-31

**Authors:** Dèlia Yubero, Núria Brandi, Aida Ormazabal, Àngels Garcia-Cazorla, Belén Pérez-Dueñas, Jaime Campistol, Antonia Ribes, Francesc Palau, Rafael Artuch, Judith Armstrong, Working Group

**Affiliations:** 1 Department of Clinical Biochemistry and Institut d’Investigació Sanitària Sant Joan de Déu, Hospital Sant Joan de Déu, Barcelona, Spain; 2 Facultat de Medicina, Universitat de Barcelona, Barcelona, Spain; 3 Molecular and Genetics Medicine Section, Hospital Sant Joan de Déu, Barcelona, Spain; 4 Department of Neurology, Hospital Sant Joan de Déu, Barcelona, Spain; 5 Institut de Bioquímica Clínica, Hospital Clínic i Provincial, Barcelona, Spain; 6 Centro de Investigación Biomédica en Red de Enfermedades Raras (CIBERER), Instituto de Salud Carlos III, Barcelona, Spain; Baylor Research Institute, UNITED STATES

## Abstract

**Background:**

Next-generation sequencing (NGS) technology has allowed the promotion of genetic diagnosis and are becoming increasingly inexpensive and faster. To evaluate the utility of NGS in the clinical field, a targeted genetic panel approach was designed for the diagnosis of a set of inborn errors of metabolism (IEM). The final aim of the study was to compare the findings for the diagnostic yield of NGS in patients who presented with consistent clinical and biochemical suspicion of IEM with those obtained for patients who did not have specific biomarkers.

**Methods:**

The subjects studied (n = 146) were classified into two categories: Group 1 (n = 81), which consisted of patients with clinical and biochemical suspicion of IEM, and Group 2 (n = 65), which consisted of IEM cases with clinical suspicion and unspecific biomarkers. A total of 171 genes were analyzed using a custom targeted panel of genes followed by Sanger validation.

**Results:**

Genetic diagnosis was achieved in 50% of patients (73/146). In addition, the diagnostic yield obtained for Group 1 was 78% (63/81), and this rate decreased to 15.4% (10/65) in Group 2 (*X*^2^ = 76.171; p < 0.0001).

**Conclusions:**

A rapid and effective genetic diagnosis was achieved in our cohort, particularly the group that had both clinical and biochemical indications for the diagnosis.

## Introduction

Inborn errors of metabolism (IEM) comprise heterogeneous and rare genetic diseases with a variety of overlapping or unspecific clinical phenotypes. Multiple proteins with enzymatic, transporter, regulatory and other functions participate in the intricate complexity of metabolic pathways. A breakdown of the normal function of some of these proteins may impair the metabolic state of an organism. These disruptions can generally be assessed biochemically through the detection of metabolites in different biological fluids. However, the specificity and sensitivity of some of these biomarkers are not always high. IEM are, generally, severe diseases, and the accurate identification of the molecular basis of these diseases is important for appropriate patient treatment and genetic counselling.

The establishment of an IEM diagnosis is supported by clinical suspicion and biochemical investigations. The classic molecular studies previously conducted in clinical laboratories consisted of time-consuming and expensive genetic studies. Some genetic diseases are complex, for example, one gene can be associated with different phenotypes, and thus, similar phenotypes may be caused by mutations in different genes. In addition, the inheritance patterns of some of these diseases are not always completely characterized. Currently, next-generation sequencing (NGS) technology has arisen as an essential tool for rapid and effective diagnoses prior to complex functional studies (i.e. enzyme activities in cells). However, due to the nature of IEM and the uncertainties of NGS, the need for a holistic approach to IEM diagnosis remains unambiguous. To date, the NGS diagnostic applications that have been published have focused on specific disorders and overlapping phenotypes, but the appearance of a clinical exome strategy has facilitated the simultaneous assessment of different phenotypes [1, 2]. With respect to IEM, studies of hyperphenylalaninemia, phenylketonuria [3], cerebral creatine deficiency [4], glycogen storage diseases [5], and mitochondrial diseases [6] have generallyyielded good results. Most studies utilized whole exome sequencing (WES), but customized NGS approaches are being currently implemented in clinical practice [7, 8, 9, 10]. Additionally, the comprehensive genetic testing of Mendelian childhood diseases through next-generation sequencing is cost-saving [11].

In the present work, we studied 146 patients with a clinical suspicion of an IEM through targeted gene sequencing (a customized panel) to establish a definitive genetic diagnosis. The strategy involved the study of a set of 171 genes and an assessment of the clinical and biochemical data that led to the selection of the patients with the aims of comparing the diagnostic yields among different clinical groups, and assessing the technical and biological limitations of this type of study regarding its potential for diagnostic application in hospital laboratories.

## Materials and Methods

### Subjects

One-hundred and forty-six patients with a clinical suspicion of an IEM were selected (all of the patients were younger than 18 years). In all cases, prior to the genetic study, various biochemical markers were studied in our laboratory according to the clinical suspicion of IEM. To avoid diagnostic bias, only patients with a non-genetic diagnosis were recruited for the study, and a period of only 2.5 years from the first to the last genetic capture performed in our laboratory (7^th^ March 2013 and8^th^ October 2015) was considered. Other diagnoses that were not within this period or were studied in other centers using different molecular approaches were not included in this study.

According to their clinical and biochemical suspicion, the patients were classified into different nosological groups, including aminoacidopathies, organic acidurias, free fatty acid oxidation defects, neurometabolic defects and complex molecules metabolism defects (which included sterol metabolism defects, glycogen storage and carbohydrate diseases, and lysosomal and peroxisomal disorders). To compare the diagnostic yield obtained after the NGS approach, the patients were classified into two groups depending on the level of evidence supporting the suspicion and independently of the NGS analysis results: Group 1 (n = 81) included patients who presented with consistent clinical and biochemical data supporting their having an IEM studied in the panel (S1 Table), and Group 2 (n = 65) included cases with clinical suspicion of IEM but without specific or impaired biochemical markers or with unexpected biochemical findings after metabolic testing. The patients were studied by experienced pediatric neurologists and clinical biochemists. A finding that the phenotype fit the descriptions of the different diseases considered provided clinical evidence of the disease In the analysis of the biochemical data, increased or decreased concentrations of the different metabolites studied in the biological fluids were interpreted according to the current guidelines for the diagnosis of IEM [12]. The biochemical markers that are considered specific (or special assays) were subjected to the external quality control program from ERNDIM. Other biomarkers, such as histopathological or neuroimaging markers, were considered during the clinical investigation of the patients when required.

### Ethical statement

The study was approved by the Ethical Committee of the Hospital Sant Joan de Déu (HSJD), and samples from the patients and controls were obtained according to the Helsinki Declarations of 1964 as revised in 2001. Written informed consent for genetic testing was obtained from all the patients or their parents/guardians.

### Samples

For the biochemical screening, blood, urine and cerebrospinal fluid (CSF) samples were collected and analyzed depending on the clinical suspicion. For the genetic studies, genomic DNA was extracted from blood leukocytes using standard procedures.

### Biochemical procedures

Urinary amino acids, organic acids, glycosaminoglycans (quantitative and qualitative), purines and pyrimidines, oligosaccharides, creatine/guanidinoacetate, plasma/serum amino acids, acylcarnitines, sterols, very-long-chain fatty acids, and CSF neurotransmitters and pterins were analyzed according to standard procedures (liquid and gas chromatography with mass spectrometry detection, HPLC with diode array, fluorescence and electrochemical detection and spectrophotometric procedures) [13]. The non-specific assays included the analyses of glucose, cholesterol, ammonia, uric acid, aminotransferases, urinary reducing agents, vitamins and other molecules.

### Genetic analysis

One hundred and seventy-one genes, which were selected according to several criteria, were targeted for the diagnosis of IEM. The first criterion was the presence of a biochemical marker in the body fluids and constituted the first-line investigations of the disease. Second, different nosological groups were defined according to clinical suspicion and included diseases with no (or unspecific) biochemical markers in the biological fluids that presented a suggestive clinical phenotype. S1 and S2 Tables show the IEM genes analyzed in the patients and their corresponding MIM numbers. A definitive genetic diagnosis was considered when previously reported pathogenic mutations were identified [according to the human gene mutation database (HGMD® Professional 2015.4)]. To evaluate putative pathogenicity of novel mutations ACMG guidelines [14] were followed; familial segregation *in silico* pathogenicity prediction (PolyPhen-2, SIFT) and frequency [Minor Allele Frequency (MAF), CIBERER Spanish Variant Server (CSVS-BIER)] criteria were analyzed in combination with the above-mentioned biochemical markers. The inheritance of all of the patients with genetic findings was checked regarding their progenitors, and the findings were validated by Sanger DNA sequencing (S2 Table).

A custom-panel design was prepared using Sure Design software (Agilent Technologies) for the HaloPlex Target Enrichment System and Illumina platform based on the last genome build available (H. sapiens, hg19, GRCh37, February 2009) and by selecting for a 150 base-pair read length. The target parameters were the coding exons and the UTRs, including a region extension of 25 bases from the 3’ end and 25 bases from the 5’ end (based on RefSeq database). The stringency parameter was selected to maximize the coverage. The step-by-step capture protocol is available online (HaloPlex Target Enrichment System Protocol, G9900-90001, version E.0, December 2014). Sequencing was performed using an Illumina sequencer (MiSeq), and two bioinformatics programs were concurrently used to assess the data (DNAnexus and VariantStudio). Concerning the implementation of the NGS technology and the reliability of the obtained sequences, the mean per-base coverage depth for the designed panels was 355-fold, and 93% of the bases were covered by more than 20-fold. The complete NGS data of all the studied samples are available at the Sequence Read Archive (http://www.ncbi.nlm.nih.gov/sra; study accession number SRP073359).

Specific oligonucleotides were designed to validate all of the candidate variants found in the patients and to study the exonic regions that were not covered in some of the genes (S1 Table). The progenitors and other family members were also studied to evaluate the inheritance model. The PCR products were purified using Exo-Sap (Affymetrix), and the sequencing reactions for both the forward and reverse strands were performed using a BigDye Terminator v3.1 Cycle Sequencing Kit (Applied Biosystems). The analysis was performed using an ABI 3130XL analyzer (Applied Biosystems). Other techniques were applied to complete the genetic study in cases of negative or partial NGS results, and these techniques included MLPA (for *PAH* and *SLC2A1* genes: SALSA® MLPA® probemix P055, SALSA® MLPA® probemix P138-C1, respectively) and quantitative PCR analysis using oligonucleotides designed for a particular case (*OTC* gene: GoTaq®, Promega; 7500 Real-Time PCR System, Applied Biosystems).

### Statistical analysis

A Chi-square test was applied to compare the different diagnostic yields in the two groups of patients. The statistical calculations were performed using SPSS 22.0 version.

## Results

A definitive genetic diagnosis was achieved for 73 out of the 146 patients (50%) (S2 Table). In addition, for 12 of 146 (8.2%) patients, we found only one variant (in diseases with a recessive inheritance pattern) or variants of uncertain significance in the candidate genes studied. No mutations in the analyzed genes were detected in 61 of 146 (41.8%) patients. Eight of these 61 patients presented clinical and biochemical data strongly suggestive of an IEM, and thus, other approaches, such as a search for mutations not detected by NGS through cDNA studies, quantitative PCR and functional studies, were used for the diagnosis. For the other 53 cases, because clinical suspicion was not supported by biochemical data, the results were considered negative.

The diagnostic yield for Group 1 was 78% (63 out of 81), and this rate decreased to 15.4% (10 out of 65) in Group 2 (Chi-square = 76.171; p < 0.0001). Ten of the cases in Group 1 were further studied because one pathogenic mutation or changes with controversial biological effects on the protein were detected. These were PKU (n = 7), glycine encephalopathy (n = 1) and free fatty acid oxidation defects (n = 2: VLCAD and MCAD) (S2 Table). No mutations were found in eight patients, including Hartnup disease (n = 1), PKU (n = 2), PNPO (n = 1), lysosomal storage diseases (n = 2), SLOs (n = 1) and peroxisomal disorders (n = 1). Concerning Group 2, the 10 positive cases included glycogen storage diseases (n = 2), lysosomal storage diseases (n = 5), neurometabolic conditions (n = 2) and a CPS1 deficiency (n = 1). In contrast, only two cases remained under study because one pathogenic mutation was detected (a glycogen storage disease and a CPTII deficiency). 53 cases were considered negative because no mutations were found.

**Fig 1:**
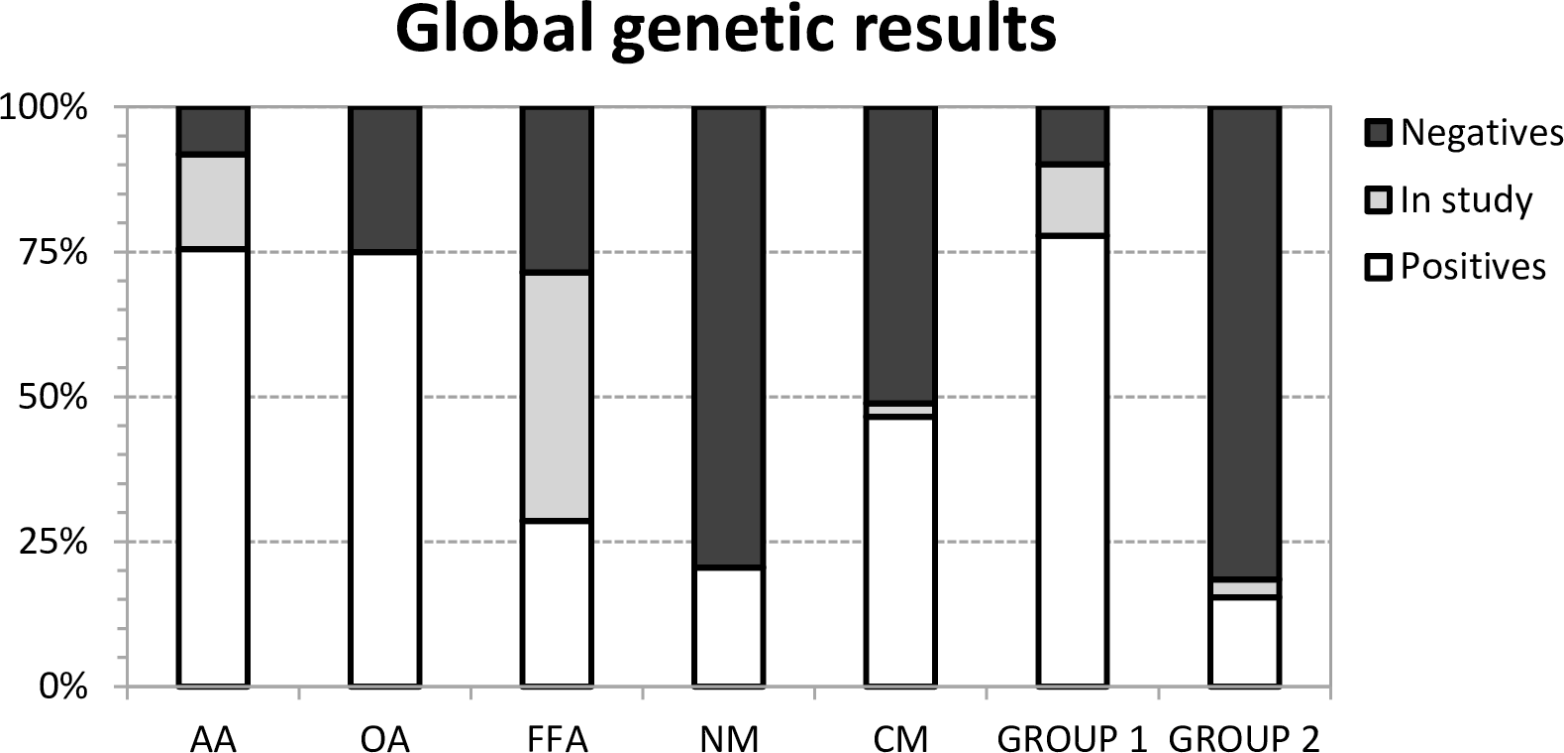
Global genetic results. Genetic results (positive, under-study, and negative cases) shown as a percentages for each nosological group (aminoacidopathies (AA); organic acidurias (OA); free fatty acid oxidation defects (FFA); and neurometabolic (NM) and complex molecules (CM) defects) and for both diagnostic groups (Groups 1 and 2).

The genetic results (diagnosed, undiagnosed and under-study patients) considering the nosological groups and diagnostic groups (groups 1 and 2) are illustrated in Fig 1, which shows the group of aminoacidopathies and organic acidurias displaying the highest diagnostic yield.

## Discussion

After application of the NGS approach in our laboratory, the diagnosis rate increased remarkably. Distinct NGS strategies are available to address genetic diagnosis of IEM, which include the combination of classical sequencing with more or less broad NGS studies. In a practical point of view, due to the high number of genes involved and the low number of patients for each single disease, we selected the most convenient methodology, which clearly accelerated certain diagnostics and reduced the costs. IEM offers the opportunity to take advantage of the availability of biomarkers that support the choice among all of the detected genetic findings. Biochemical diagnosis must remain a key part of the diagnostic algorithm to ensure that the selected genetic variants are the real cause of the observed phenotype because most of these workflows are still biased by personal experience and lack of specificity. Unsolved cases can be explained by various reasons. First, the causative gene might not be included in the panel design. There may be genes encoding proteins involved in the alteration of a specific biochemical marker that are currently unknown or not related with human disease. Second, metabolic diseases are highly heterogeneous, and overlapping phenotypes might confuse the clinical orientation. In these cases, the disease-causative gene could be involved in another pathway but could yield a similar phenotype. Ultimately, methodology limitations are critical. A fraction of the coding regions remain unsequenced, and thus, false negatives are the largest problem that we confront in the genetic diagnosis, even though the false-negative and false-positive rates are lower than those obtained in WES and WGS studies [6]. Moreover, we might miss some variations, as our strategy was not designed for the detection of deep intronic or regulatory mutations [15, 16] and it does not reach MLPA accuracy to detect copy number variants (CNVs).

### Group 1: patients with specific biochemical findings

In this study, we investigated a large cohort of patients with different IEM. The patients in Group 1 displayed a high rate of definitive genetic diagnosis, supporting the hypothesis that clinical assessment in combination with consistent biochemical markers is an indispensable tool for the diagnosis of IEM patients. Regarding unsolved cases, some patients remain under investigation as only mutations in one allele were found, and some other were considered negatives as we found no pathogenic variants. The most plausible explanation for the patients with partial findings is due to the selected methodology limitations. In PKU, the *PAH* gene has more than 800 reported mutations, and some of these are located in deep intronic regions that are not covered by our technical genetic design. The mutation detection rate for PKU is high [17, 18, 19, 3]. Although the existence of patients with incomplete molecular findings is relatively frequent [17], different NGS approaches are capable of increasing the diagnostic yield to 100% [3]. An explanation for free fatty oxidation defects, may be that the carrier status appears to be the most common finding in large cohorts of VLCAD patients [20], and moreover, 30% of the patients had no genetic diagnosis, because no variants were detected in the *ACADVL* gene. An unusual case was a patient with a classical NKHG (based on the clinical presentation and biochemical data, including liver enzyme activity), without mutations in any of the three genes directly involved in the glycine cleavage system, only a variant of uncertain significance. In previous reports, no mutations in NKHG-related genes were found in approximately 5% of the patients with enzyme-proven GCE [21]. Moreover, defects in lipoate biosynthesis and the related iron sulfur cluster biogenesis genes (*LIAS*, *BOLA3* and *GLRX5*) were found to be causative for some patients with GCE without disease-causing mutations in the genes encoding the glycine cleavage enzyme [21]. Thus, functional studies of this patient should be performed to assess the impact of this variant and explore lipoylation defects.

Some diseases belonging to Group 1 enclosed negative cases. For instance, one of the patients had a first-step diagnosis of Hartnup disease. The causative gene for Hartnup, *SLC6A19* [22], encodes the transporter B0AT1, and to date, no mutations have been found in the studied families [23]. Two accessory proteins (Tmem27 and ACE2) interact with the neutral amino acid transporter [24, 25], but their link to the etiology of the disease is unknown. Therefore, even though the characteristic amino acid profile in urine points to Hartnup disease, more investigations need to be performed to completely comprehend the biology and pathophysiology of amino acid transporters. The patient with a suspicion of PNPO deficiency presented neonatal epilepsy associated with a profound CSF PLP deficiency, increased CSF amino acids and normal biogenic amines. Thus, we cannot rule out the possibility that this PLP deficiency may be secondary to other genetic or even non-genetic causes. In the four cases with complex molecules defects, the biochemical markers impairment detected were an increase in urinary GAG excretion or an increase in 7-dehydrocholesterol and very-long-chain fatty acids in serum. This group of patients, are being subjected to further biochemical and genetic testing to definitively confirm or rule out the presence of an IEM.

### Group 2: patients with non specific biochemical findings

In this group, even though diagnostic yield was low (15.4%), the ten diagnosed cases are a good example of the utility of NGS technology because the diagnosis of these cases through the conventional Sanger approach would have been very time consuming. A good example was a 12-year-old girl with attention deficit and hyperactivity who presented with hyperammonemia and increased glutamine values. The genetic testing performed revealed two unexpected mutations in the *CPS1* gene (S2 Table). The two neurometabolic conditions (GLUT1 and DAT defects, S2 Table) showed mild or normal biomarker results. For the seven cases with glycogen or lysosomal storage diseases, neither impaired biomarkers nor biochemical data were available in addition to the clinical suspicion. The 53 negative cases from Group 2 mainly belonged to neurometabolic disorders, sterol metabolism and lysosomal storage diseases, reflecting the non-specificity of (or lack of) the existing biomarkers for some of these IEM patients, which added a level of complexity to the diagnosis. For example, the worst outcome regarding the genetic diagnosis was observed for the neurometabolic patients. The explanation for this heterogeneous group may be relatively simple. The biomarkers available are not specific, and we promoted both the clinical and biochemical phenotype in the selection criteria. For instance, because the growing descriptions for a GLUT-1 deficiency include cases with normal or near-normal CSF glucose values, the patients under suspicion of this syndrome were selected based on their clinical phenotype [26, 27]. In the analysis of biogenic amines, impaired CSF HVA concentrations are associated with a large amount of neurological conditions, as previously reported [28], or cerebral folate deficiency syndrome, encompassing a large group of neurological conditions in which partial deficiencies unrelated to the primary defects in folate transport and metabolism are frequently encountered [29]. Despite the fact that epileptic encephalopathies are a frequent phenotype observed in neurometabolic disorders, IEM are not the most frequent etiology causing this neurological disruption. However we consider of importance to study a possible metabolic cause for these disorders, essentially because some of these are potentially treatable conditions. However, probably the best strategy to face neurometabolic disorders would be to start with a broader approach, such as clinical exome.

In conclusion, clinical assessments in combination with, consistent biomarkers are useful tools for increasing the diagnostic yield in IEM patients. In these cases, the use of targeted gene panels investigated by NGS is highly productive and cost-effective. The patients with no specific biomarkers represent more complexity. However, even if further clinical investigation is needed, the NGS panel approach yields important results and may help distinguish those patients who require further investigation with an additional exome/genome sequencing approach.

## Supporting Information

S1 Table List of genes included in the design organized by nosological group and associated disease. Columns 3 and 4 show the RefSeq NM number used for the analysis of each of the genes and the percentage of the covered region.
(XLSX)

S2 Table Positive (white background) and inconclusive (grey background) cases. The mutation and protein effects for each case are represented (* = reported mutation). Inheritance was studied based on case availability (1 = 1 progenitor tested; 2 = both progenitors tested; nt = inheritance not tested). A definitive diagnostic decision was obtained depending on the specific laboratory tests (special assays), and in the absence of biomarker cases, a diagnosis was achieved directly through molecular genetics and using *in silico* prediction software (ASSP = Alternative Splice Site Predictor; PolyPhen-2).
(XLSX)
